# Antibacterial and Antibiofilm Efficacy and Mechanism of Ginger (*Zingiber officinale*) Essential Oil against *Shewanella putrefaciens*

**DOI:** 10.3390/plants12081720

**Published:** 2023-04-20

**Authors:** Chi Zhang, Yao Xie, Weiqiang Qiu, Jun Mei, Jing Xie

**Affiliations:** 1 College of Food Science and Technology, Shanghai Ocean University, Shanghai 201306, China; 2 Key Laboratory of Aquatic Products High Quality Utilization, Storage and Transportation (Co-Construction by Ministry and Province), Ministry of Agriculture and Rural Affairs, Shanghai 201306, China; 3 National Experimental Teaching Demonstration Center for Food Science and Engineering, Shanghai Ocean University, Shanghai 201306, China; 4 Shanghai Engineering Research Center of Aquatic Product Processing and Preservation, Shanghai 201306, China; 5 Shanghai Professional Technology Service Platform on Cold Chain Equipment Performance and Energy Saving Evaluation, Shanghai 201306, China

**Keywords:** ginger essential oil, active compounds, antibacterial mechanism, cell membrane destruction

## Abstract

Ginger (*Zingiber officinale*) has unique medicinal value and can be used to treat colds and cold-related diseases. The chemical composition and antibacterial activity of ginger essential oil (GEO) against *Shewanella putrefaciens* were determined in the present study. Zingiberene, α-curcumene, and zingerone were the main active compounds of GEO. GEO displayed significant antibacterial activity against *S. putrefaciens,* with a minimum inhibitory concentration (MIC) and minimum bactericidal concentration (MBC) of 2.0 and 4.0 μL/mL, respectively. Changes in intracellular ATP content, nucleic acid and protein structure, exopolysaccharides (EPS) content, and extracellular protease production indicated that GEO disrupted the membrane integrity of *S. putrescens*. At the same time, changes in biofilm metabolic activity content and the growth curve of biofilm showed that GEO could destroy the biofilm. Both scanning electron microscopy (SEM) and confocal laser scanning microscopy (CLSM) observations confirmed that GEO destroyed the cell membrane and lead to the leakage of the constituents. The above results indicate that GEO entered the cells via contact with bacterial membranes, and then inhibited the growth of *S. putrefaciens* and its biofilms by increasing membrane permeability and inhibiting various virulence factors such as EPS. The findings showed that GEO could destroy the structure of cell membrane and biofilm of tested *S. putrefaciens*, indicating its potential as a natural food preservative.

## 1. Introduction

*Shewanella putrefaciens* is a Gram-negative bacteria and a specific spoilage organism in low-temperature storage aquatic products [1]. *S. putrefaciens* is the dominant spoilage bacterium commonly found in aquatic products, which can contribute to food spoilage and the erosion of food handling equipment, leading to severe health issues and significant economic losses [2]. The saprophytic bacteria can reduce trimethylamine N-oxide to trimethylamine [3]. Moreover, *S. putrefaciens* can produce H_2_S (the main source of fishy smell) through protein and amino acid metabolism [4]. The existence of biofilm is the most important factor in the resistance of *S. putrefaciens* to drugs and extreme environmental resistance [5]. In microbial communities, the adhesion of cells to aquatic products inevitably undergoes a transition from a planktonic to an aggregated state, resulting in the formation of biofilms [6]. Biofilms usually exhibit a three-dimensional structure consisting of a matrix (proteins, polysaccharides, DNA and other components) secreted by cells [7]. Biofilms can serve as a natural obstacle that promotes metabolic variations [8], enables microbes to adjust to slow growth, and makes them more resistant to preservatives [9]. Once a biofilm forms on the surface of aquatic products and equipment, it is difficult to remove. Even with stringent disinfection methods, safety concerns caused by biofilms still arise [10].

In recent years, essential oils (EOs) have been added to food as natural preservatives in order to extend the shelf life of food [11]. EOs are complex mixtures of aromatic and volatile secondary metabolites of aromatic plants, usually obtained from plant materials such as flowers, fruits, and leaves [12]. Some EOs are known to fight pathogenic bacteria, such as *Melissa officinalis* L. essential oil [13]. Recent studies have shown that the essential oils of *A. scoparia*, *A. absinthium* [14], *Tagetes minuta* L. [15], and *Buddleja asiatica* [16] essential oil have a significant inhibitory effect on the cell membrane of Gram-negative bacteria. Ginger (*Zingiber officinale*) has a unique acrid aroma, so it can be used as a spice in food [17]. Moreover, ginger contains many biological compounds, such as zingiberene, 6-gingerol, and α-curcumene [18]. Ginger essential oil is a volatile oil drawn from roots. Ginger essential oil (GEO) has application potential in the drug, cosmetics, and food fields [19]. Moreover, in 2018, GEO received generally recognized as safe (GRAS) status by the Food and Drug Administration (FDA) [20].

The antibacterial mechanisms of GEO against *Bacillus subtilis* [21], *Staphylococcus aureus* [22], and *Escherichia coli* [23] have been reported. Nevertheless, few authors have studied the antibacterial activity and mechanism of GEO against *S. putrefaciens*. Therefore, this study investigated the inactivation of *S. putrefaciens* planktonic and biofilm cells by GEO and examined its antibacterial and antibiofilm mechanisms.

## 2. Results

### 2.1. Essential Oil Composition Analysis by Gas Chromatography-Mass Spectrometry (GC-MS) Results

The composition of GEO was determined by GC-MS [24], and 16 major constituents were identified (Table 1). The major bioactive components of GEO were zingiberene, α-curcumene, and zingerone. Among them, zingiberene is the main compound, accounting for 34.4% of the total. α-curcumene constituted 13.7% of the and was the second volatile component in GEO, followed by zingerone (10%). These results were in line with the results of the study by Wang et al. [1]. Al-Dhahli et al. also found that zingiberene and α-curcumene were the main components of GEO [2].

### 2.2. Results of Antibacterial Activity

#### 2.2.1. Minimum Inhibitory Concentration (MIC) and Minimum Bactericidal Concentration (MBC)

The antibacterial activities of GEO against *S. putrefaciens* were quantified by the results of MIC and MBC values. The MIC and MBC values of *S. putrefaciens* were 2.0 ± 0.125 ^a^ and 4.0 ± 0.257 ^a^ µL/mL, respectively (*p* < 0.05) (The same lowercase letters indicate that the difference is not significant).

#### 2.2.2. Integrity of Cell Membrane

**Figure 1:**
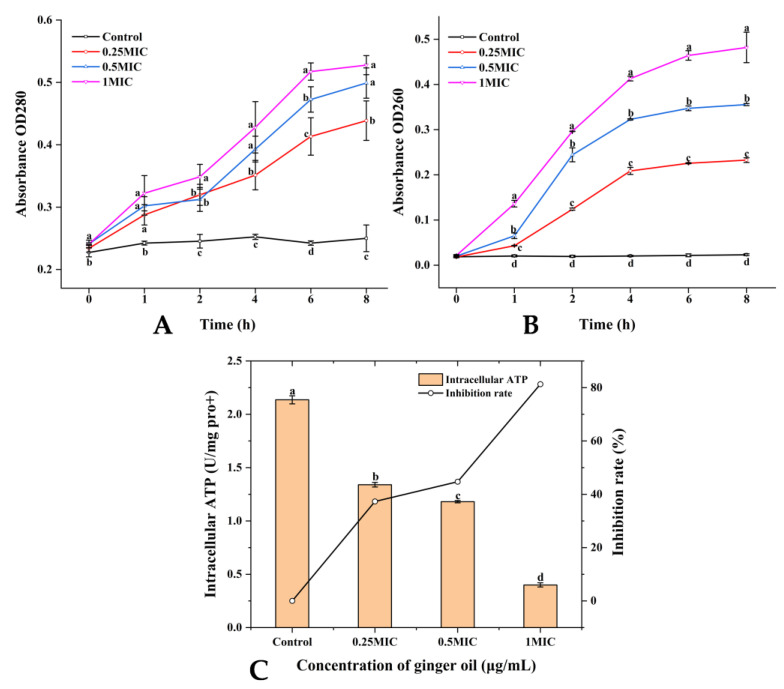
Analysis of (**A**) Nucleic acids level, (**B**) Protein level, and (**C**) Intracellular ATP. (**A**,**B**) express changes in nucleic acids and proteins of *S. putrefaciens* over time and in response to changes in GEO concentration. (**C**) represents the changes of intracellular ATP content in *S. putrefaciens* with the change in GEO concentration. Different colors in (**A**,**B**) indicate different concentrations (Black—Control, Red—0.25 MIC, Blue—0.5 MIC, Violet—1 MIC). Different lowercase letters indicate significant difference (*p* < 0.05).

The release of proteins and nucleic acids from *S. putrefaciens* was significantly increased by the GEO treatment. In Figure 1A,B (*p* < 0.05), the number of extracellular proteins and nucleic acids also rose with concentration, and this effect was time-dependent. The leakage of nucleic acids was slow during 1–2nd h, accelerated from the 2nd h to the 6th h, and leveled off after 6th h. Unlike protein, the leakage rate peaked during the 1st h to 4th h and became slow after the 4th h.

#### 2.2.3. Intracellular ATP

The changes in intracellular ATP levels of *S. putrefaciens* cells after being treated with GEO are shown in Figure 1C. The intracellular ATP levels of the control were maintained at 2.14 U/mg pro+. The intracellular ATP was 1.34 and 1.18 U/mg pro+ for 0.25× and 0.5× MIC, respectively. The intracellular ATP level (0.4 U/mg pro+) of the GEO treated samples decreased significantly (*p* < 0.05). The intracellular ATP values of the 1× MIC GEO treated samples were markedly lower than those of the control (*p* < 0.05), indicating that the damage levels of the 1× MIC GEO cell membrane were higher than for the other samples.

#### 2.2.4. Fourier Transform Infrared (FTIR) Spectroscopic Analysis

**Figure 2:**
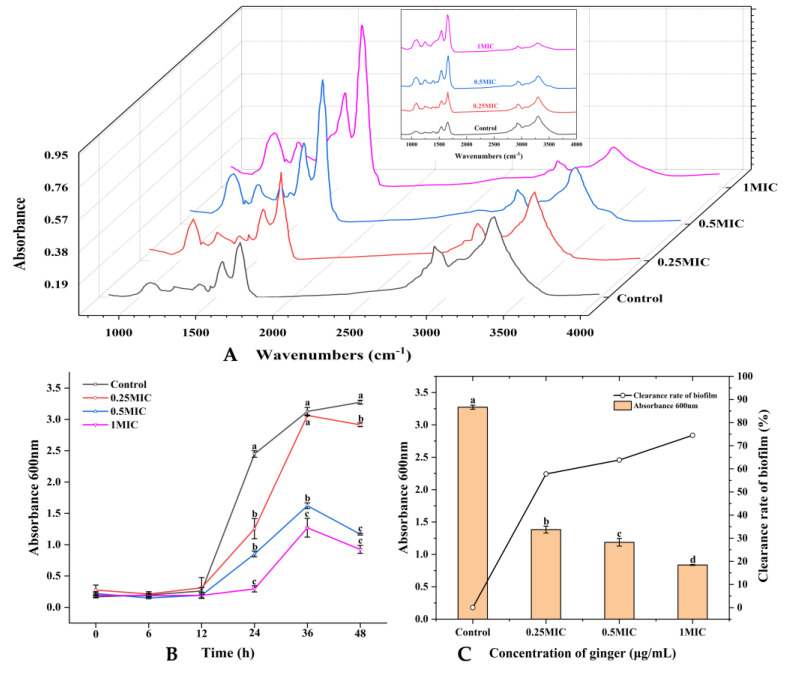
Analysis of (**A**) FTIR, (**B**) the growth curve of biofilm (**C**) removal activity. (**A**) expresses the changes produced in FTIR by *S. putrefaciens* with the change of GEO concentration. (**A**) indicates the growth curves of biofilms of *S. putrefaciens* at different GEO concentrations. (**C**) indicates the ability of different concentrations of GEO to eliminate the biofilms of *S. putrefaciens*. Different colors in (**A**,**B**) indicate different concentrations (Black—Control, Red—0.25 MIC, Blue—0.5 MIC, Violet—1 MIC). Different lowercase letters indicate significant difference (*p* < 0.05).

The macromolecules of *S. putrefaciens* changed significantly (*p* < 0.05) (Figure 2A). The absorption peak at 3288 cm^−1^ represents the functional group of lipids; the absorption peak at around 1244 cm^−1^ represents the phosphodiester associated with the phospholipid bilayer; the absorption peaks at 1626 and 1543 cm^−1^ represent the functional group of protein: the absorption peak of 1626 cm^−1^ represents the amide I in the α-helical structure, the absorption peak at 1543 cm^−1^ represents the N–H bond of protein amides, and the absorption peaks at 1070 cm^−1^ and 1082 cm^−1^ represent the functional groups of nucleic acids. The absorption peak intensities at 3288 cm^−1^ (lipid) and 1244 cm^−1^ (phosphodiester) of *S. putrefaciens* after being treated with GEO were significantly weakened and enhanced compared with the control (*p* < 0.05). The intensities of the absorption peak at 1626 cm^−1^ and 1543 cm^−1^ increased significantly (*p* < 0.05).

### 2.3. Results of Antibiofilm Activity

#### 2.3.1. Growth Curve of Biofilm

Different concentrations of GEO inhibited the biofilm formation activities of *S. putrefaciens*. The growth curve of the biofilm is shown in Figure 2B. The growth rate of biofilm was slow during 0–12 h. At 12th h, the amount of biofilm (expressed in absorbance) in the control group was 0.262, for 0.25× MIC it was 0.313, for 0.5× MIC it was 0.192, and for 1× MIC it was 0.191. The biofilm of *S. putrefaciens* grew rapidly from the 12th to 36th h. The amount of biofilm in the essential oil treatment group also reached its peak; the biofilm amount of the 0.25× MIC treatment group was 3.062, the amount in the 0.5× MIC treatment group was 1.622, and the amount in the 1× MIC treatment group was 1.27. From the 36th h to the 48th h, the biofilm started to break up, and new biofilm progressively developed. It was obvious that the biofilms treated with GEO ruptured faster at this time, and the formation rate of new biofilms decreased significantly (*p* < 0.05).

#### 2.3.2. Removal Activity

The removal of *S. putrefaciens* biofilm by different concentrations of GEO can be seen in Figure 2C. As the GEO concentration increased, the OD value kept decreasing, which indicated that the biofilm kept decreasing. The GEO displayed a positive biofilm removal influence on *S. putrefaciens*. When the GEO concentration was 0.25× MIC, the biofilm achieved a percentage removal of 57.76%. At a concentration of 1× MIC, the removal rate was as high as 74.45%.

#### 2.3.3. Inhibition of Motility by GEO

**Figure 3:**
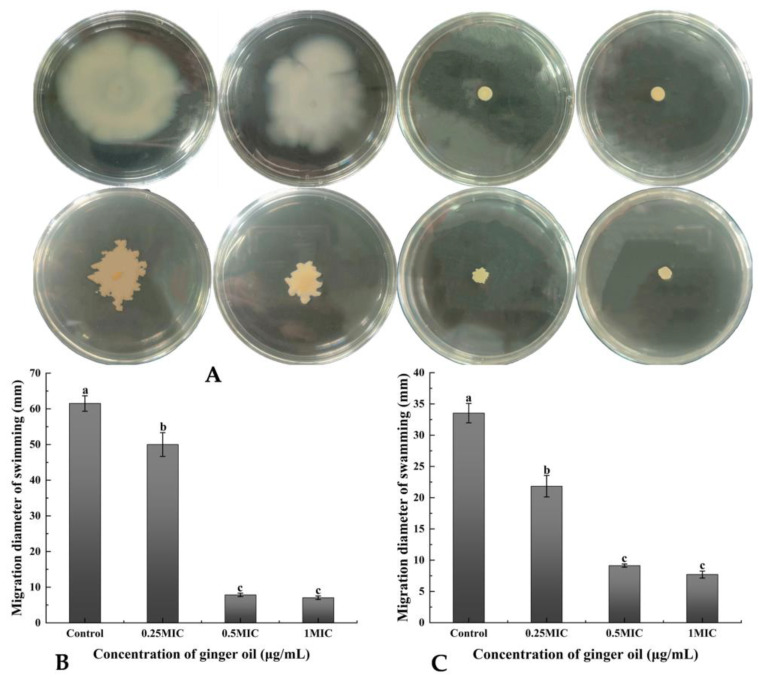
Analysis of (**A**) swimming and swarming photography, and the migration diameter of (**B**) swimming and (**C**) swarming. (**A**) indicates the growth curves of biofilms of *S. putrefaciens* at different GEO concentrations. (**A**–**C**) indicate changes in the motility of biofilms of *S. putrefaciens* with the change in GEO concentration. Different lowercase letters indicate significant difference (*p* < 0.05).

The inhibition of *S. putrefaciens* motility is a strategy for potentially inhibiting biofilm formation. The present study showed that GEO inhibited the motility of *S. putrefaciens* in a dose-dependent manner on swimming and swarming plates (Figure 3A–C). The swimming motility of *S. putrefaciens* was significantly inhibited with 0.5× and 1× MIC (*p* < 0.05). Therefore, GEO inhibited the biofilm generation of *S. putrefaciens* through suppressing its capacity to access the bottom layer. At 0.25× MIC, the bacterial colonies became smaller, and the inhibition of swimming movement was not obvious enough, but the colony morphology of the surge plate was no longer uniform, and the colony diameter decreased from 61.5 mm to 50 mm in the control group. At 0.5× and 1× MIC, the swimming movement was substantially inhibited with the shape of a small dot in the center of the plate, and the colony diameter was 8.2 mm and 7.1 mm. In addition, the GEO also had a positive effect on the control of the swarming movement. The 0.25×, 0.5× and 1× MIC GEO all inhibited the swarming movement of the bacteria. The colony diameter of the control group was 33.5 mm, and the colony showed an uneven shape. At 0.25× MIC, the colony became smaller, and the colony diameter was 21.83 mm. At 0.5 and 1× MIC, the colony diameter was drastically reduced, reaching 9.13 mm and 7.7 mm.

#### 2.3.4. Inhibition of GEO on Exopolysaccharides (EPS) Production

**Figure 4:**
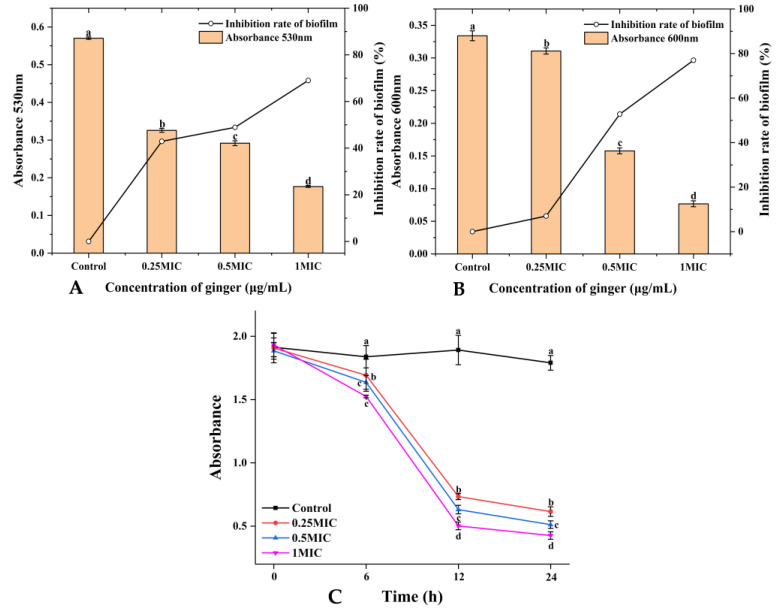
Analysis of (**A**) EPS production, (**B**) extracellular protease production, and (**C**) metabolic activity. (**A**,**B**) indicate the changes in EPS and the extracellular protease of *S. putrefaciens* biofilms in response to changes in GEO concentration. (**C**) indicates the changes in metabolic activity of *S. putrefaciens* biofilms with time and GEO concentration. Different colors in C indicate different concentrations (Black—Control, Red—0.25 MIC, Blue—0.5 MIC, Violet—1 MIC). Different lowercase letters indicate significant difference(*p <* 0.05).

As shown in Figure 4A, 0.25× and 0.5× MIC GEO evidently inhibited the EPS level in *S. putrefaciens* biofilms. The EPS yield dropped by 42.89% and 48.86% in the 0.25× and 0.5× MIC groups. The results indicate that a low concentration of GEO had a good inhibitory influence on the production of EPS by *S. putrefaciens*. The 1× MIC GEO inhibited EPS production in *S. putrefaciens* by 69.02%. The decrease in EPS was compatible with the biofilm production results.

#### 2.3.5. Inhibition of GEO on Extracellular Protease Production

Compared with the control, the higher the concentration of GEO, the more it could inhibit the production of extracellular protease. The 0.25× MIC GEO inhibited 6.99% of extracellular protease production in *S. putrefaciens* (Figure 4B). The results showed that a low concentration of GEO could not inhibit the extracellular protease production in *S. putrefaciens*. The inhibition rate of *S. putrefaciens* extracellular protease production by 1× MIC GEO was 77.04%.

#### 2.3.6. Biofilm Metabolic Activity

As indicated in Figure 4C, the metabolic activity of the control was not reduced. However, the metabolic activity of the samples treated with GEO decreased significantly (*p* < 0.05). The GEO did not inhibit the metabolism of *S. putrefaciens* until the sixth hour, whereas GEO could rapidly inhibit the metabolism from the 6th to the 12th h and keep the metabolic activity of *S. putrefaciens* at a low level after the 12th h. These results could be observed more visually numerically; at the 6th h, the metabolic activity of the control was 1.838, 0.25×, 0.5×, and the 1× MIC was 1.692, 1.636 and 1.525, respectively. At the 12th h, the control was 1.892, while the other three groups decreased to 0.733, 0.631, and 0.502, and remained at a lower level at the subsequent time. This is consistent with the results of EPS quantification and biofilm removal experiments. These findings indicated that the biofilm of *S. putrefaciens* was damaged by GEO, and *S. putrefaciens* lost its metabolic activity.

#### 2.3.7. Scanning Electron Microscope (SEM)

**Figure 5:**
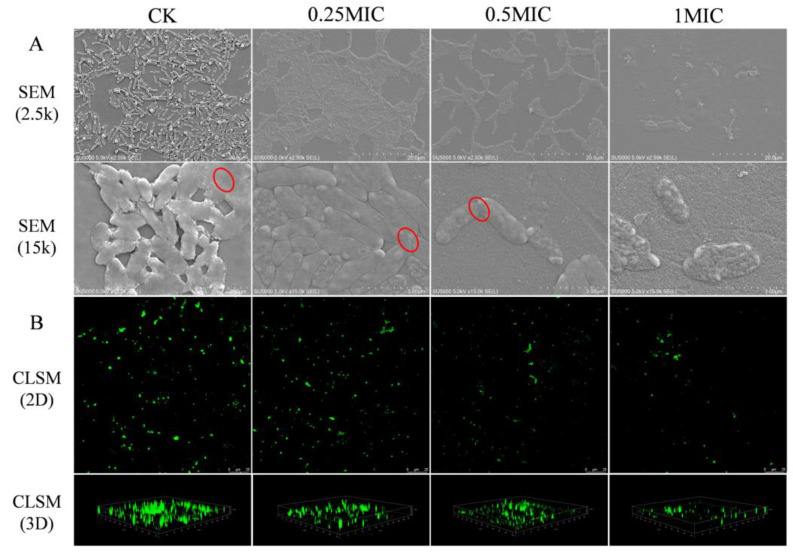
(**A**) SEM photography, (**B**) CLSM photography. A indicates the biofilms of *S. putrefaciens* at different SEM magnifications (2.5k, 15k), and the connections between biofilms are marked with red circles. (**B**) shows the CLSM images of *S. putrefaciens* biofilms in 2D and 3D.

In the control, biofilms had structurally complete cell-to-cell connections (represented by ellipses). Biofilms were able to display smooth and regular cells with complete membranes (Figure 5A). In contrast, the biofilm architecture of GEO-treated samples was broken down. The coarse and irrational exterior indicated that the GEO treated bacterial cells had lost their typical appearance. SEM images demonstrated that GEO obliterated the biofilm by destroying the intercellular junctions and invoking cell lysis.

#### 2.3.8. Confocal Laser Scanning Microscopy (CLSM)

**Figure 6:**
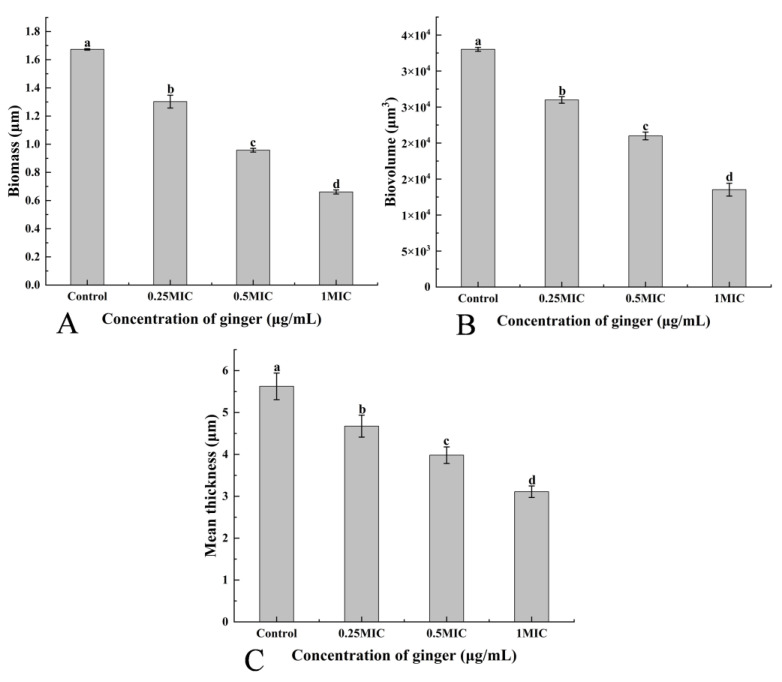
(**A**) biomass, (**B**) biovolume, and (**C**) mean thickness. (**A**–**C**) show the three CLSM indicators of biofilms at different GEO concentrations, respectively. Different lowercase letters indicate significant difference (*p* < 0.05).

The CLSM image of the biofilm is shown in Figure 5B. The biofilm of control displayed heavy and compact communities. The GEO-treated samples showed that GEO could significantly diminish the biofilm of *S. putrefaciens*, and the reduction of biomass and biovolume was proportional to the concentration of GEO (*p* < 0.05). The indicators of CLSM (Figure 6A–C) showed that the space between colonies became larger and the biofilm thickness became smaller with the increase in GEO concentration. These results demonstrated that the production of exopolymeric substrates by bacterial cells in biofilms was suppressed in the presence of GEO. This discovery was consistent with the findings gained from the EPS assay.

## 3. Discussion

*Shewanella* is a gram-negative bacteria that can cause aquatic products to be corrupted [25]. Here, the antibacterial and anti-biofilm potential of GEO was evaluated to determine whether it has the potential to attenuate the biofilm construction and other virulence factors of *S. putrefaciens*. First, the main chemical composition of GEO was determined in this study. Mesomo et al. identified that the primary feature of GEO was in the form of oxygenated monoterpenes, such as zingiberene. In fact, zingiberene and α-curcumene have been documented to have powerful antibacterial action [26]. In addition, some studies believed that the antibacterial activity of GEO came from the bioactive components (gingerol, 6-gingerol and α-Cumene). These main bioactive components of GEO could inhibit the growth of bacteria by attacking the cell membrane and cell wall [27]. After a comparison with the aforementioned study and summarizing, the main antibacterial active ingredient in GEO was determined to be zingiberene. Thongson et al. reported that GEO showed higher antibacterial activity (the MIC is 0.6 and the MBC is 2 µL/mL) against *Salmonella typhimurium* strains than the extracts of ginger [28]. Singh et al. [29] noted that GEO demonstrated a higher inhibition of *Staphylococcus aureus*, *Klebsiella pneumoniae*, *Pseudomonas aeruginosa,* and *Escherichia coli* than the oils obtained with a few solvents.

Nucleic acids and proteins are essential materials that exist in the whole cell of bacteria, and they flow out with the damage of the bacterial membrane [30]. Hence, the spillage of nucleic acids and protein is related to the membrane integrity. This may be due to the increased penetration of the cell membrane after being treated with GEO, resulting in the bacteria lacking fundamental structural capabilities, and in the release of nucleic acids and proteins from the cell to the outside. The GEO, even at a low concentration, could also destroy the cell membrane of *S. putrefaciens*. This is similar to the bactericidal effect of clove leaf essential oil studied by Zhang Yi et al. [31]. ATP has an important function in membrane transport [32]. The intracellular ATP concentration represents the injury levels of the *S. putrefaciens* cell membrane [33]. This result revealed that GEO destroyed the integrity of the *S. putrefaciens* membrane and caused ATP leakage (*p* < 0.05). Sun et al. also found that chlorogenic acid could decrease the intracellular ATP levels of *Pseudomonas fluorescens* [34]. In addition, the hydrophobic compounds of GEO bind to the phospholipid portion of the membrane and mitochondria, damaging their identity and function (enzyme activity, protein, nucleic acid and energy metabolism) [35]. The absorption peak intensities at 3288 cm^−1^ (lipid) and 1244 cm^−1^ (phosphodiester) of *S. putrefaciens* after being treated with GEO were significantly weakened and enhanced compared with the control (*p* < 0.05). This result indicated that GEO could damage the phospholipid bilayer of the cell membrane [36]. The absorption peak at 1070 cm^−1^ in the control shifted from the absorption peak at 1082 cm^−1^ after GEO treatment. It can be seen that GEO destroyed the structure of nucleic acids. The intensities of the absorption peak at 1626 cm^−1^ and 1543 cm^−1^ were significantly increased (*p* < 0.05), indicating that GEO changed the secondary structure of the membrane protein [37]. These findings were analogous with the results of the cell membrane integrity assay and of the extracellular proteases assay. FTIR can indirectly characterize the disruption mechanism of *S. putrefaciens* biofilm exposed to GEO, reflecting the change in the molecular composition of both bacteria before and after GEO treatments. GEO inhibits the development and propagation of *S. putrefaciens* by destroying cell membranes.

**Figure 7:**
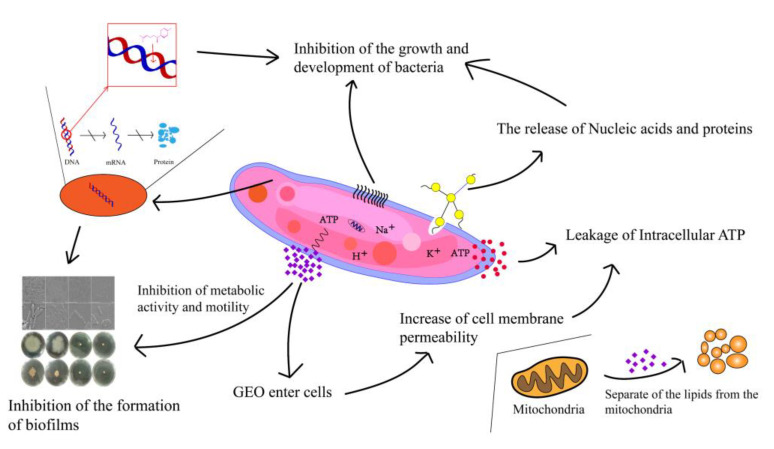
The antibacterial and antibiofilm mechanism of GEO.

Compared to planktonic cells, biofilms possess better immunity to antimicrobial agents [38]. The inhibition of biofilm generation by GEO was more pronounced in the early stage. Cui et al. [39] also reported that the biofilm of *S. aureus* was dispelled by cardamom essential oil. GEO with a high concentration of GEO could effectively remove the mature *S. putrefaciens* biofilms [13]. The possible reasons were that GEO increased the cell membrane permeability and decreased intracellular ATP levels. Millezi et al. [40] concluded that cinnamon essential oil and tea tree essential oil showed excellent removal of *E. coli* and *S. aureus* biofilms. Flagellar-driven motility (swimming and swarming) has a crucial role in the elementary association of bacteria, which spreads over the surface as a biofilm sheath [41]. Nazareth et al. also found that fruit polyols suppressed biofilm generation by disrupting the swimming motion of *P. aeruginosa* and *Yersinia enterocolitic* [42]. In addition, bee colony behavior is a key virulence factor due to its involvement in biofilm construction. GEO significantly reduced the swarming motility and the diffusion diameter of *S. putrefaciens* (*p* < 0.05). This was in agreement with the findings of Yin et al. [43], who found that soy isoflavones inhibited the swarming motility of *P. aeruginosa*. Weaker swarming movements may lead to the progression of lesser biofilms, which may have major implications for the treatment of *S. putrefacien* infections. Our results suggest that GEO has a good inhibitory effect on biofilm motility. Zhang et al. [44] also found that the motility and the biofilm generation of *Erwinia carotovora* and *P. fluorescens* were dramatically restricted by cinnamon essential oil. EPS forms the widest segment of biofilms and has multiple functions, ranging from supplying structural and mechanical stability [45]. EPS played an essential role in the original stage of biofilm formation [46]. This was also confirmed by the study of *V. parahaemolyticus* by Yu et al. Extracellular proteases are capable of digesting a wide range of host proteins directly or are indirectly involved in the processing of other toxic proteins, therefore identified as potential virulence factors [47]. This result was consistent with the cell membrane permeability, intracellular ATP and FTIR results, indicating that GEO could suppress biofilm generation by inhibiting the activity of extracellular protease. Cao et al. also found that citral could effectively inhibit the production of extracellular proteases [48]. XTT is a methyltetrazolium salt analogous to MTT [3-(4,5-dimethyl2-thiazolyl)-2,5-diphenyl-2H tetrazolium bromide]. Yellow-colored XTT can be reduced to orange-colored formazan products by live cell mitochondria [49]. Xie et al. found that chitosan could reduce the metabolic activity of *V. parahaemolyticus* [50]. Chen et al. demonstrated that borage essential oil could inhibit the metabolic activity of *Shigella flexneri* [51]. Clove essential oil could also damage the biofilm by destroying the connection between cells [52]. Our results found that GEO had the same effect. This was similar to the results of EPS production, extracellular protease production, and swarming motility. The same results were obtained by Wijesundara et al. in the study of the antibacterial mechanism of carvacrol [53]. However, 0.25× MIC GEO-treated samples still had a cell-cell junction, but the cell had been disrupted. The CLSM can observe the biological structure of the bacterial structure without affecting their component [54]. The CLSM results showed a concentration measure dependence of GEO on *S. putrefaciens*. Ashrafudoulla et al. found a similar trend when studying the effect of eugenol on *L. monocytogenes* and *P. aeruginosa* biofilms [55]. Liu et al. found that the mean thickness and biofilm formation of *E. faecalis* could be suppressed by thyme oil [56]. In particular, CLSM images of biofilms before and after treatment with thyme essential oil revealed a reduction in the cell number [57]. The antibacterial mechanism and antibiofilm mechanism of GEO against *S. putrefaciens* was shown in Figure 7. The GEO component entered the bacterial cell and disrupted the cell membrane, leading to the increased permeability of the cell membrane, resulting in the leakage of nucleic acids and proteins and ATP, thus inhibiting the growth and reproduction of *S. putrefaciens*. For biofilm, GEO reduced EPS production and extracellular protease activity by inhibiting the metabolic activity and motility of biofilm, thereby inhibiting biofilm formation, and disrupting the biofilm by disrupting the connections between biofilms. Meanwhile, the Zingiberene of GEO could bind to DNA and cause DNA damage, thus inhibiting the transcription process of *S. putrefaciens*. DNA damage resulted in the insufficient synthesis of ATP, EPS and other substances essential for the growth of bacteria and biofilms, thus inhibiting the growth of bacteria and biofilms [58].

## 4. Materials and Methods

### 4.1. Materials

GEO was acquired from Macklin Biochemical Co., Ltd. (Shanghai, China). *S. putrefaciens* CICC 24940 was applied in the present research. An amount of 100 μL of the original cultures were inoculated in 10 mL of TSB media with 3% NaCl (*w*/*v*) (Hopebio, Qingdao, China). The cultures were multiplied at 37 °C by shaking at 200 r/min to obtain the primary cultures of 10^8^ CFU/mL. The GEO was dissolved with 10% Tween-80 (*v*/*v*) and then ultra-sonicated for 3 min until fully dissolved.

### 4.2. Essential oil Composition Analysis by Gas Chromatography-Mass Spectrometry (GC-MS)

GEO measurements were performed using GC-MS (Trace DSQ II, Thermo Fisher Scientific, Cambridge, MA, USA). A HP-5ms (30 mm × 250 μm × 0.25 μm) capillary fused silica column was used. In terms of temperature setting, the syringe was 250 °C and the detector was 230 °C. The ion source was 230 °C. The oven temperature was 60 °C, and it was raised to 220 °C to heat the injector at the rate of 20 °C/min after 2 min. The injection sample volume was 1 µL, the ionization energy was 70 EV, and the scan range was 35–500 *m*/*z*. The GEO was analyzed and identified by matching the retention times in a mass spectra library. The retention index was determined using the n-alkanes C7–C30 [19].

### 4.3. Antibacterial Activity Assay

#### 4.3.1. MIC and MBC Determination

The MIC and MBC of *S. putrefaciens* treated with GEO were obtained by the micro-dilution according to Dai et al. [59]. MIC is the lowest concentration of GEO in the well plate where no bacterial growth can be seen [60]. MBC was expressed to the minimum GEO concentration for the growth of a sterile colony on the culture dish [61]. The bacterial culture (10^8^ CFU/mL) was added to a 96-well plate. Consecutive dilutions of GEO were produced. The solutions (0.125 to 256 µL/mL) were then added to every well. Next, all plates were cultured at 37 °C for 24 h, and the growth was evaluated using a turbidimetric test. The cultures from MIC conditions were passaged on a medium to identify MBC without colony growth [62].

#### 4.3.2. Integrity of Cell Membrane Determination

GEO with the concentration range (1×, 0.5×, 0.25× and 0× MIC) were mixed with the primary culture, and the mixture was cultured for 4 h at 37 °C with shaking. The samples were then centrifuged (8000× *g*, 4 °C, 10 min) and the supernatant was collected. Nucleic acids and protein levels in the collected supernatant were monitored by their OD_260_ and OD_280_ (UV1900i, SHIMADZU, Kyoto, Japan) [63].

#### 4.3.3. Intracellular ATP Determination

The concentrations of ATP in the *S. putrefaciens* were determined according to Song et al. [32]. *S. putrefaciens* was treated with GEO (1×, 0.5×, 0.25× and 0× MIC) for 30 min, and the acquired culture (1 mL) was mixed with PBS (0.01 mol/L, 9 mL) and centrifuged (10,000× *g*, 1 min, 4 °C). The supernatants were then obtained to determine the concentrations of ATP according to the colorimetric method.

#### 4.3.4. Fourier Transform Infrared Spectroscopic Analysis

After centrifuging (8000× *g*, 10 min), the mixture with bacterial cultures and GEO was collected and cleaned twice with PBS (0.01 mol/L), and the precipitation was fixed in a 2.5% glutaraldehyde solution for 4 h and cleaned with PBS (0.01 mol/L) three times. The precipitates were then gathered and freeze-dried for 48 h. Finally, an FTIR spectroscopy was performed (Nicolet, Thermo Fisher Scientific, Cambridge, MA, USA) [64]. The FTIR spectroscopy was obtained from the wavenumber range (4000–400 cm^−1^).

### 4.4. Antibacterial Activity Assay

#### 4.4.1. Biofilm Formation Determination

*S. putrefaciens* was cultured (37 °C, 24 h) and incubated (37 °C, 24 h) at a dilution of 1:200 (final volume of 2000 μL) in plates. After incubation, the suspension was then removed, fresh LB broth (Huan Kai Microbial, Guangzhou, Guangdong, China) was added to each well, and the suspension was inoculated (37 °C for 24 h). After that, the suspension was removed again, and fresh LB broth was added and inoculated at 37 °C for 24 h.

#### 4.4.2. Biofilm Biomass Determination (Crystal Violet Staining Assay)

The influence of GEO on biofilm was evaluated with crystal violet staining [65]. First, GEO with the concentration ranges (1×, 0.5×, 0.25×, and 0× MIC, respectively) was mixed with the original culture and cultured for 48 h. The culture was then inoculated into another plate and cultured for 24 h. The suspended cells were then removed, and 200 μL of medium containing GEO (1×, 0.5×, 0.25× and 0× MIC, respectively) was added and cultured for 24 h. The liquid was finally removed,, cleaned twice with 0.01 mol/L PBS, dried, and then dyed with crystal violet [66]. The absorbance of the solution was measured at 570 nm.

#### 4.4.3. Growth Curve Determination of Biofilm

According to the biomass measurement method in Section 4.4.2, the biomass of biofilm was measured at 6, 12, 24, 36 and 48 h to draw the growth curve of biofilm.

#### 4.4.4. Motility Assay

The influence of GEO on movement performance measures, namely swimming and swarming, was evaluated. For the swarming test, 5 µL *S. putrefaciens* cultures (1 × 10^8^ CFU/mL) was then added to LB plates with 1.5% (*w*/*v*) agar. The GEO (1×, 0.5×, 0.25× and 0× MIC, respectively) was added and incubated at 37 °C for 24 h. For the swimming motility assay, 5 µL bacterial cultures (1 × 10^8^ CFU/mL) were added to the center of the LB plates with 0.3% (*w*/*v*) agar with GEO (1×, 0.5×, 0.25×, and 0× MIC, respectively), and incubated at 37 °C for 12 h. Finally, the shape and spread diameter of the colonies on the petri dish were observed.

#### 4.4.5. EPS and Extracellular Protease Determination

The EPS was determined by the phenol sulfuric acid method. The cultures were centrifuged (2500× *g*, 15 min). The supernatants were mixed with three times the volume of 95% (*v*/*v*) ethanol and left at 4 °C for 24 h. The samples were then centrifuged (2500× *g*, 15 min). The precipitate was then gathered and combined with the mixture of water, phenol (5%, *v*/*v*), and concentrated sulfuric acid in the ratio of 1:1:5 (*v*:*v*:*v*) and reacted for 20 min. The EPS levels were then calculated by absorbance measurements at 490 nm [56].

Changes in the extracellular protease levels of *S. putrefaciens* were examined based on the study by Cao et al. [48]. In short, overnight cultures of *S. putrefaciens* were cocultured for 9 h by inoculating the LB broth (1 × 10^5^ CFU/mL) with different concentrations (1×, 0.5×, 0.25× and 0× MIC) of GEO. The proceeds were centrifuged at 14,000× *g* for 1 min, and the supernatant was then gathered by incorporating an equivocal amount of PBS containing 10 mg/mL Hide Powder Azure (HPA, St. Louis, MO, USA) and incubated at 37 °C for 2 h. Next, 2 mL of 10% (*v*/*v*) trichloroacetic acid was added to stop the reaction. The extracellular protease levels were quantified by registering the absorbance at 600 nm.

#### 4.4.6. Biofilm Metabolic Activity Determination (XTT Reduction Assay)

The XTT test was conducted to determine the strength of the metabolic activity of *S. putrefaciens* [67]. The XTT labeling reagent and electron coupling reagent were melted in a water bath at 37 °C, followed by mixing at the proportion of 50: 1 as an XTT reagent. After the treatment procedure shown in the biomass assay, 100 μL PBS and 50 μL XTT reagent were mixed in each well and cultivated without light at 37 °C for 24 h. In the end, absorbance was determined at 450 nm using a microplate reader (Multiskan FC, Thermoscientific, Cambridge, MA, USA).

#### 4.4.7. SEM assay

Cultures were centrifuged at (1700× *g*, 4 °C, 10 min) to gather biofilm. The biofilm of *S. putrefaciens* were fixed in a 2.5% glutaraldehyde solution for 4 h and cleaned with 0.01 mol/L PBS solution [68]. Finally, the *S. putrefaciens* were eluted with the gradient ethanol solutions (10%, 30%, 50%, 70%, 90%, 100%, respectively), and the bacterial culture was observed by SEM (S-3400, Hitachi, Tokyo, Japan) [69].

#### 4.4.8. CLSM Assay

CLSM (LSM710, Carl Zeiss AG, Oberkochen, Batenfurt, Germany) was performed to investigate the influence of GEO on biofilm. The excitation wavelength was 488 nm, and the emission wavelength was 525 ± 25 nm. After 24 h of biofilm agglutination, the supernatant was eliminated, and the plates were cleaned three times with PBS (0.01 mol/L) [70]. The samples were fixed in glutaraldehyde, and the plates were cleaned with PBS (0.01 mol/L). Samples were stained to avoid light using SYBR Green I (Sangon Biotech, Co., Ltd., Shanghai, China) for 30 min before observation. Finally, Biomass, biovolume and mean thickness were determined by COMSTAT 2 software [71].

### 4.5. Statistical Analysis

All of the experiments were performed in three independent repeats [72]. Data were analyzed on SPSS 26.0 software using ANOVA with a Duncan’s test and expressed as mean ± standard deviation (SD). Different lowercase letters (a–d) indicate statistically significant differences (*p* < 0.05). *p* < 0.05 was considered as the significant level for all statistical tests. Origin 2021 was used for graph construction.

## 5. Conclusions

This research investigated the antibacterial and antibiofilm activities of GEO against *S. putrescens* and its mechanism. GEO demonstrated intense antibacterial activity against *S. putrescens* with an MIC and MBC of 2.0 and 4.0 μL/mL, respectively. GEO broke the phospholipid of the membrane, causing nucleic acids and protein leakage. XTT and crystal violet assays showed that GEO disrupted the biofilms by reducing biomass and cellular activity. Both SEM and CLSM images revealed physical injury, substantial ethological changes, and the leakage of intracellular components. GEO reduced the amounts of biofilm, destroyed the biofilm structure, and delayed movement. According to the above results, GEO has a promising potential to be used as a natural food preservative and to promote a longer shelf-life of food. Future studies are expected to delineate additional mechanisms underlying the anti-biofilm behavior of GEO.

## Figures and Tables

**Table 1:** Analysis of volatile components of ginger essential oil.

No.	Retention Index	Compounds	Formula	Percentage (%)
1	971	Zingiberene	C_15_H_24_	34.4
2	916	α-Curcumene	C_15_H_22_	13.7
3	974	Zingerone	C_11_H_14_O_3_	10
4	899	Santalol	C_15_H_24_O	4.2
5	887	1,6,10-Dodecatriene,7,11-dimethyl-3-Methylene-, (6E)-	C_15_H_24_	4.1
6	1136	Geraniol	C_10_H_18_O	1.4
7	788	Dipentene	C_10_H_16_	1.4
8	803	Benzaldehyde	C_7_H_6_O	0.91
9	572	(+)-borneol	C_10_H_18_O	0.65
10	805	Linalool	C_10_H_18_O	0.53
11	889	Trans-Cinnamaldehyde	C_9_H_8_O	0.52
12	828	Beta-Phellandrene	C_10_H_16_	0.44
13	895	Caryophyllene	C_15_H_24_	0.41
14	904	Cinnamaldehyde	C_9_H_8_O	0.41
15	906	(−)-isolongifolene	C_15_H_24_	0.39
16	903	(E, Z)-2,6-dimethylocta-2,4,6-triene	C_10_H_16_	0.37

## Data Availability

And on reasonable request, the corresponding author will provide the data used or analyzed during this investigation.
